# A novel enterocyte-related 4-gene signature for predicting prognosis in colon adenocarcinoma

**DOI:** 10.3389/fimmu.2022.1052182

**Published:** 2022-12-02

**Authors:** Xuehua Cheng, Yong Wei, Yugang Fu, Jiacheng Li, Li Han

**Affiliations:** ^1^ Department of Traditional Chinese Medicine (TCM) Geriatrics, Huadong Hospital Affiliated to Fudan University, Shanghai, China; ^2^ Translational Medicine Department, GeneScience Pharmaceuticals Co. Ltd., Changchun, China; ^3^ Shanghai Municipal Hospital of Traditional Chinese Medicine, Shanghai University of Traditional Chinese Medicine, Shanghai, China

**Keywords:** colon adenocarcinoma, scRNA-seq, immunotherapy, signature model, prognostic biomarkers

## Abstract

**Background:**

Colon adenocarcinoma (COAD) is a fatal disease, and its cases are constantly increasing worldwide. Further, the therapeutic and management strategies for patients with COAD are still unsatisfactory due to the lack of accurate patient classification and prognostic models. Therefore, our study aims to identify prognostic markers in patients with COAD and construct a cell subtype-specific prognostic model with high accuracy and robustness.

**Methods:**

Single-cell transcriptomic data of six samples were retrieved from the Gene expression omnibus (GEO) database. The cluster annotation and cell-cell communication analysis identified enterocytes as a key player mediating signal communication networks. A four-gene signature prognostic model was constructed based on the enterocyte-related differentially expressed genes (ERDEGs) in patients with COAD of the Cancer Genome Atlas cohort. The prognostic model was validated on three external validation cohorts from the GEO database. The correlation between immune cell infiltration, immunotherapy response, drug sensitivity, and the four-gene signature prognostic model was investigated. Finally, immunohistochemistry (IHC) was performed to determine the expression of the four genes.

**Results:**

We found that the proportion of epithelial cells was obviously large in COAD samples. Further analysis of epithelial cells showed that the activity of the enterocytes was highest in the cell-cell communication network. Based on enterocyte data, 30 ERDEGs were identified and a 4-gene prognostic model including *CPM*, *CLCA4*, *ELOVL6*, and *ATP2A3* was developed and validated. The risk score derived from this model was considered as an independent variable factor to predict overall survival. The patients were divided into high- and low-risk groups based on the median riskscore value. The correlation between immune cell infiltration, immunotherapy response, immune status, clinical characteristics, drug sensitivity, and risk score was analyzed. IHC confirmed the expression of signature genes in tissues from normal individuals, patients with polyps, and COAD.

**Conclusion:**

In this study, we constructed and validated a novel four-gene signature prognostic model, which could effectively predict the response to immunotherapy and overall survival in patients with COAD. More importantly, this model provides useful insight into the management of colon cancer patients and aids in designing personalized therapy.

## Introduction

Colon adenocarcinoma (COAD) is the most common type of colon cancer and the second most prevalent cancer worldwide (1). Aging, genetic mutations, alcohol consumption, smoking, unhealthy dietary habits, obesity or being overweight, and hereditary are some of the risk factors for COAD. There has been a significant reduction in the incidences of COAD due to an increase in screening for colon cancer; however, the mortality rate is expected to rise by 60% by 2035 (2, 3). It has been well established that colon cancer arises from the glandular epithelial cells of the colon. Under normal physiological conditions, the structure of the glandular epithelial cells is the same as normal cells and is responsible for absorption and digestion. However, under pathological conditions, changes occur in the shape of glandular epithelial cells and undergo uncontrolled proliferation, ultimately leading to the development of colon cancer.

Previous studies have performed bulk sequencing of the tumor sample to study the transcriptomic profile of patients with COAD, which provides information on the gene expression pattern in all cells, but they fail to capture the heterogeneity of tumor cells. Single-cell sequencing technology helps overcome these limitations and provides a deeper coverage, thereby enhancing our understanding of the heterogeneity of COAD. The profiling of different subpopulations of tumor cells aids in unraveling the heterogeneity exhibited by these cells during the development and progression of COAD. Deciphering the precise cellular composition and developmental trajectory of COAD has helped identify novel genes associated with pathogenesis and underlying mechanisms for the malignant transformation of COAD (4). Moreover, the single-cell sequencing technology has allowed us to explore the immune microenvironment of COAD, which will aid in identifying potential targets for immunotherapy for the treatment of COAD (5).

Current treatment strategies for COAD include polypectomy, radiotherapy, chemotherapy, and targeted therapy (6); however, these options are insufficient to satisfy the clinical demands. Hence, new treatment strategies like biological, cell, and gene therapy, cancer vaccines, nutritional supplement, and even combination therapy were eventually developed (7). Immunotherapy is one of the novel treatment strategies which has revolutionized cancer treatment. Immune checkpoint inhibitors (ICI) like pembrolizumab and nivolumab, which target programmed cell death 1, have been effective in the treatment of patients with metastatic colorectal cancer (CRC) (8). Studies have shown that identifying biological markers which can predict the patients’ responses to ICI treatment would aid in identifying patients who would benefit from ICI therapy (9). However, resistance to ICI monotherapy in patients with COAD could be the underlying cause of the low response rate of ICI therapy in clinical settings (10). Therefore, there is an urgent need to identify a single or combination of prognostic biomarkers which can precisely classify patients and help design personalized treatment plans.

Therefore, our study aimed to construct a prognostic model by integrating bulk and single-cell RNA sequencing (scRNA-seq) data of patients with COAD retrieved from publicly available databases. Three external validation cohorts were used to confirm the robustness of the four-gene signature prognostic model. In addition, we determined the ability of the prognostic model to predict distinct immune characteristics, response to ICIs treatment, and drug sensitivity. Finally, IHC was used to study the expression pattern of the four signatures. Our results provide potential prognostic biomarkers and therapeutic targets for the treatment of COAD.

## Materials and methods

### Data source and acquisition

The GSE201348 dataset was retrieved from the Gene Expression Omnibus (GEO) database (http://www.ncbi.nlm.nih.gov/geo/), which includes scRNA-seq data from two normal individuals, two patients with polyps, and two patients with COAD. The data from 23,770 cells were used in this study. Bulk transcriptomic data and the relevant clinical information of 473 patients with COAD were retrieved from The Cancer Genome Atlas (TCGA; https://www.tcga.org/). The GSE14333, GSE10347, and GSE72970 datasets retrieved from the GEO database was used as three external validation cohorts. The demographic information of patients with COAD is shown in **Supplementary Table 1**.

### scRNA-seq data processing

The Seurat R package was used to perform the scRNA-seq analysis. All parameters were set to default if not specifically listed. The percentage of erythrocyte and mitochondrial genes was calculated during quality control to remove low-quality cells. The “FindVariableFeatures” function filtered the top 2000 highly variable features, and the cells were normalized using the “ScaleData” function. Principal component analysis (PCA) and Uniform Manifold Approximation and Projection (UMAP) algorithms were used for dimensionality reduction and visualization. Harmony (v1.0) was used to eliminate batch effects between all samples. The “FindAllMarkers” function was used to annotate the clusters based on the typical cell markers.

### Identification of epithelial cell subtypes, cell-cell communication, and pseudotime analysis

The epithelial cell subtypes were identified based on the expression of marker genes as described previously (11). The “CellChat” R package was used to study cell-cell communication by calculating the intensity of interaction of cell-cell communication based on the law of mass interaction in combination with expression profiles of known ligands, receptors, and their cofactors (12). Finally, the “monocle2” package was used to study the single-cell trajectory, and the “DDR-Tree” was used for pseudo-time analysis.

### Identification and functional analysis of differentially expressed genes

DEGs from six samples were divided into different groups. Group 1 consisted of DEGs from normal individuals and patients with polyps, and group 2 consisted of patients with polyps and patients with COAD. Venn diagram was used to identify overlapping DEGs between groups 1 and 2 and calculated using the “linear models for microarray data” (limma) R package. The DEGs were screened based on the cutoff criteria: False Discovery Rate< 0.05 and |log2FC| > 1. These overlapping DEGs were called enterocyte-related differentially expressed genes (ERDEGs). To quantify the functional pathway activity of ERDEG-associated cancer markers, gene set variation analysis (GSVA) was performed in both groups of patients. Pathway analysis was performed primarily on signature channels described in the Molecular Signature database, which were exported using the Gene Set Enrichment Analysis (GSEA) Base package. We also used the described dataset to assess metabolic pathway activities. To assign pathway activity estimates to individual cells, GSVA was applied by the standard procedure as implemented in the GSVA package.

### ERDEGs were used to construct the prognostic risk model

The prognostic significance of every differentially expressed ERDEG was assessed using the survival package’s “univariate coxph” function, and then the characteristics of the training cohort with p values< 0.05 were defined as survivor-related. Least Absolute Shrinkage and Selection Operator (LASSO)-Cox regression analysis was used to identify the most optimal gene combinations for the construction of the prognostic risk model. Ten-fold cross-validation was used to determine the optimal values of the penalty parameter *l.* LASSO COX regression analysis was used to calculate the coefficient at gene expression levels, and the formula used to calculate the riskscore was as follows:


. $$Risk\;score\;=\sum^{}expr_{i}*coef_{i}$$

In the above formula, “coef” represents the coefficient of the selected genes by the multivariate Cox regression analysis and “expr” refers to the gene expression value.

Univariate and multivariate Cox regression analyses were used to assess whether the prognostic risk model was independent of other clinicopathological parameters like age, gender, tumor location, and stage. The receiver operating characteristic (ROC) curve and the Kaplan-Meier survival curves by “survminer” and “survival” R packages were used to determine the predictive efficacy of the prognostic model. Next, we compared the prediction power of the signature with additional clinical characteristics, including gender, age, and tumor classification obtained from TCGA database to verify the model.

### Internal and external validation of the four-gene prognostic risk model

The patients with COAD from TCGA database, i.e., the internal validation cohort, were divided into high- and low-risk groups based on the median risk score value. In addition, the patients with COAD from the GSE14333, GSE10347, and GSE72970 datasets were used as external validation cohorts to verify the predictive power of the signature in the same manner as in the previous section. The batch effects were carefully eliminated for further analysis.

### Prognostic risk scores related to immune cell infiltration and immunotherapy response

The tumor mutation burden and tumor immune dysfunction and exclusion (TIDE) score were used to evaluate the ability of the four-gene signature prognostic risk model to predict the potential immunotherapeutic response. The data on immune checkpoint inhibitor (ICI) related genes like *PD-L1, LAG3*, and CTLA4, and tumor mutation data of the patients with COAD were downloaded from TCGA database and were analyzed using the “maftools” R package. The somatic copy number alteration burden was determined in fractions by genomic alterations. TIDE is a computational method that mimics two main mechanisms of tumor immune escape, i.e., T cell dysfunction and T cell rejection. TIDE scores are used to predict the patient’s response to ICI therapy. The data on TIDE scores (T cell dysfunction and T cell rejection scores) were retrieved from the online database using the link: http://tide.dfci.harvard.edu.

### Establishment and evaluation of the prognostic nomogram

The multivariate Cox regression analysis was used to construct the prognostic nomogram based on factors associated with the patient’s survival, i.e., age and tumor stage, for predicting 1-, 3-, and 5-year survival in the patients with COAD. The “rms” R package was used to map the nomogram. The prediction accuracy of the nomogram was evaluated by C-index and calibration curve.

### Drug sensitivity analysis between the high- and low-risk groups

The “pRRophetic” R package was used to distinguish the difference in drug sensitivity between the patients in the high- and low-risk groups. The relevant active ingredients were selected later based on the score calculated.

### Immunohistochemistry (IHC) analysis

IHC was performed on the tissues to determine the expression of CPM, ELOVL6, ATP2A3, and CLCA4. The anti-CPM (1:200 dilution, abs118904-50µl), anti-ELOVL6 (1:200 dilution, abs113191-50µl), and anti-ATP2A3 (1:200 dilution, abs113191-50µl) were purchased from Absin. The polyclonal anti-CLCA4 was purchased from Novus (NBP1-86688). IHC was performed, and the slides were scored as described previously (13).

### Tissue collection

Surgically excised specimens of COAD, paraneoplastic, and polyp tissues were immediately immersed in 10% neutral formalin(pH 7.2–7.4) for fixation 24h. After ethanol gradient dehydration, xylene transparency, and mounting, samples were embedded in paraffin and processed into 4µm paraffin pathological sections. The patient who did not receive chemotherapy, radiation therapy, or any pre-operative treatment were enrolled in the study.

### Statistical analysis

For continuous variables, the Wilcoxon signed-ranked test was used to compare the difference between the two groups. Cox and LASSO-Cox regression analysis was used for predictable variables. The Kaplan-Meier survival curves were used to identify the difference in the survival of patients in different risk groups. The logrank test was used to study the statistical difference. A two-sided p< 0.05 was considered statistically significant. The statistical analyses were performed using the R package (ver. 4.1.3).

### Ethics statement

All studies involving human participants were reviewed and approved by the Ethics Committee of Huadong Hospital, affiliated with the Fudan University (Shanghai, China; No.2019K069).

## Results

### Clustering of scRNA-seq data and cell type characteristics

**Figure 1:**
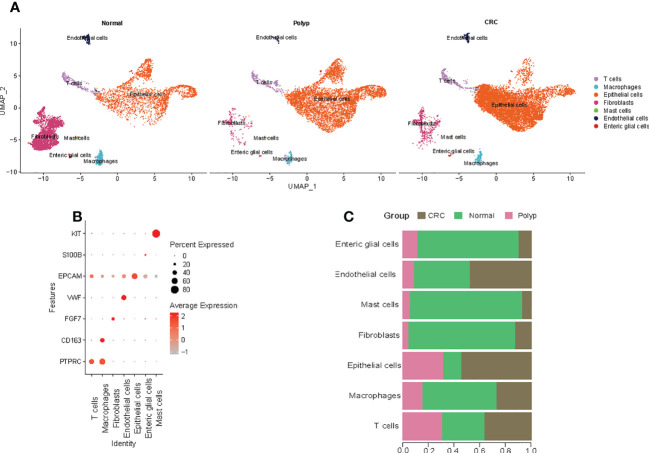
Cell types in the colon tissue from normal individuals, patients with polyps, and adenomas. **(A)** The UMAP plots of cell types from six samples. **(B)** Dot plot of the proportion of cells in the respective cluster expressing selected marker genes. **(C)** The bar plot shows the fraction of cells originating from the different samples.

A refined single-cell dataset was obtained after performing normalization and quality control on 23770 single cells obtained from six samples, including 6677 cells from normal samples, 6520 cells from polyp samples, and 10573 cells from COAD samples. A total of 22716 DEG genes were identified from this refined single-cell dataset. PCA was performed to reduce the dimensionality using the top 2000 DEGs. The UMAP algorithm was used to perform unbiased clustering of the cells. A total of seven clusters of cells were identified, including T cells, macrophages, epithelial cells, fibroblasts, mast cells, endothelial cells, and enteric glial cells (**Figure 1A**). The statistical quantification matched with biological annotations demonstrated differences in the expression of marker genes of the cell populations (**Figure 1B**). Interestingly, higher proportion of epithelial cells were observed in the patients with COAD compared to normal individuals and those with polyps (**Figure 1C**). Hence, epithelial cell subgroups were further analyzed.

### Identification of epithelial cell subtypes, cell-cell communication, and pseudotime analysis

**Figure 2:**
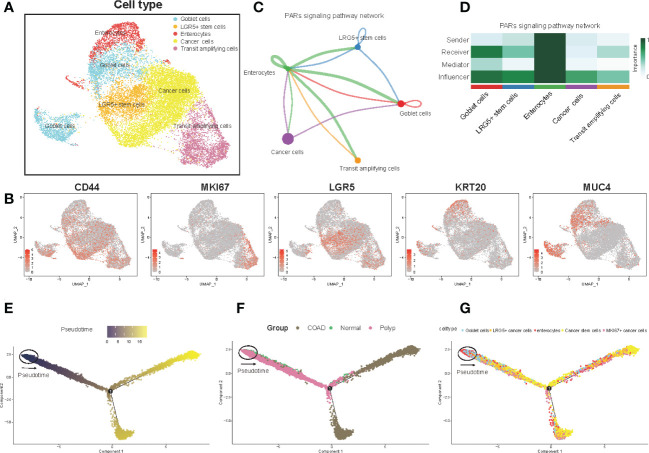
Epithelial cell clusters, cell-cell communication, and pseudotime analysis. **(A)** The UMAP plot of epithelial cells is color-coded by their associated cluster or cell classification. **(B)** Each UMAP plot shows the expression of a known cell-type specific marker gene, as indicated at the top. **(C)** The cell-cell communication network **(D)** Heatmap shows the correlation between different cell types. **(E–G)** Trajectory reconstruction **(E)** of all single cells from different samples **(F)** and cell types **(G)** is shown.

Next, the UMAP algorithm was used to identify the epithelial cells subgroup for exploring the changes in the gene signature of the prognostic risk model at the single-cell level. A total of seven clusters were identified. The clusters with identical cell types were merged, and five cell types, including goblet cells, LRG5+ stem cells, enterocytes, cancer cells, and transit amplifying cells, were identified (**Figure 2A**, **Supplementary Table 2**). **Figure 2B** shows the expression of marker genes. In addition, we evaluated the cell-cell communication network based on the specific pathways and ligand receptors. The communication probability was calculated, and the results showed that the PARs signaling pathway plays an important role in the communication network (**Figure 2C**). A heatmap was drawn to visualize the calculated probability of different components of the signaling pathway, and the results showed that the rank of the enterocytes was the highest in the PARs signaling pathway (**Figure 2D**). Finally, we investigated the overall differentiation and development of various cell subsets (**Figure 2E**). As the disease progressed, the pseudotime analysis revealed that the epithelial cells differentiated from normal cells to polyps to COAD. The goblet cells and the enterocytes responded at the start, whereas the LRG5+ stem cells and transit amplifying cells mainly responded at the end stage (**Figures 2F, G**). Overall, the enterocytes were highly active in the communication network and played an important role in the initial differentiation of epithelial cells.

### Identification and functional analysis of DEGs

**Figure 3:**
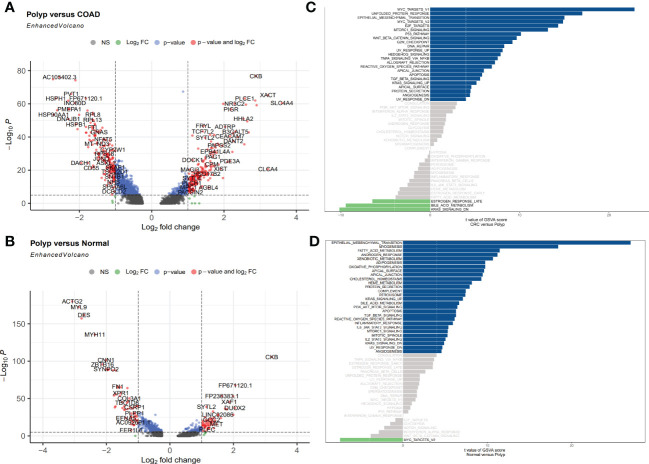
**(A, B)** Volcano plot shows upregulated and downregulated DEGs in enterocytes between different groups. **(A)** Polyp versus COAD. **(B)** Polyp versus Normal. **(C, D)** GSVA was used to determine the differences in pathways enriched per cell in enterocytes isolated from different samples. **(C)** Polyp versus COAD. **(D)** Polyp versus Normal.

Here, the DEGs between three types of samples were identified, with an emphasis on the enterocytes. Based on the predetermined cutoff values, 23 signaling pathways were significantly enriched in the patients in the polyps group compared to normal individuals, and 28 signaling pathways were significantly enriched in the patients in the polyps group compared to the patients in the COAD group. Most signaling pathways enriched were associated with well-known oncogenic pathways. A total of 376 DEGs, including 197 upregulated and 179 downregulated genes, were identified between the patients in the polyp groups and the COAD groups. Among these genes, the most significantly upregulated genes were *CLCA4, XACT*, and *SLC4A4*, whereas *PGGHG, HSP90AA1*, and *HSPH1* were the most significantly downregulated genes (**Figure 3A**). A total of 117 DEGs, including 57 upregulated genes and 60 downregulated genes, were identified between the patients in the polyp groups and tissues from normal individuals. *CKB, FP671120.1*, and *DUOX2* were the most significantly upregulated genes, whereas *TAGLN, MYL9*, and *ACT2* were the most significantly downregulated genes (**Figure 3B**). GSVA was used to explore the functional pathway enriched by various cell types and the difference in all pathways enriched by the patients in the COAD and polyp groups. MYC targets, unfolded protein response, and epithelial-mesenchymal transition (EMT) were upregulated, whereas the late response to estrogen, bile acid metabolism, and the KRAS signaling DN pathway was downregulated in the patients in the COAD group (**Figure 3C**). Compared to normal individuals, EMT, myogenesis, fatty acid metabolism, and androgen response were upregulated, whereas the MYC target pathway was downregulated in the patients in the polyp group (**Figure 3D**).

**Figure 4:**
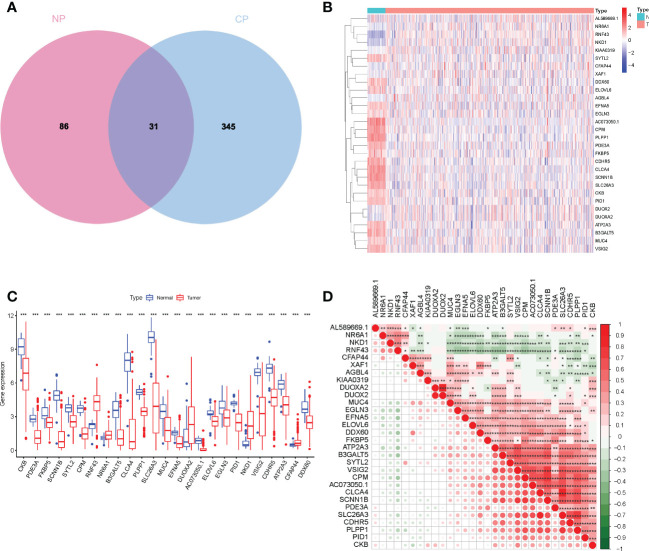
**(A)** Venn diagram to show ERDEGs between groups. **(B)** Heatmap of the expression of the 30 ERDEGs in tissues from patients with COAD and normal individuals of TCGA cohort. **(C)** A significant difference in the expression of ERDEGs in the colon tissues from patients with COAD and normal individuals. **(D)** Interaction analysis among the 30 ERDEGs. *P< 0.05, **P< 0.01, ***P< 0.001.

Consistent with previous studies, our results indicate that a few normal components of the daily diet, like estrogen and phytoestrogen, could confer protection against CRC (14). Estrogen is regulated by estrogen receptors (ER) like ESR1 and ESR2. Loss or mutation in *ESR2* is associated with the presence of colorectal polyps, tumor stage, and grade (15). In addition to fatty acid metabolism, a reduction in levels of dietary methyl donors can inhibit the development of COAD to some extent, especially sensitive to the uptake of dietary methyl donors (16). As shown in **Figure 4A**, the overlap between the two datasets with 31 DEGs was termed the enterocyte-related differentially expressed genes (ERDEGs).

To investigate the biological functions of ERDEGs and their significance in enterocyte cells, we analyzed the expression patterns of 30 ERDEGs (one of which was not expressed in the TCGA database) in 473 patients with COAD and 41 normal individuals retrieved from TCGA database. A difference in the expression of 30 ERDEG was observed in patients with COAD and normal individuals (**Supplementary Table 3**). These results suggest that the ERDEGs play an important role in tumor occurrence and progression. The correlation among 30 ERDEGs was evaluated to investigate the nature of the interaction between ERDEGs. A significant positive correlation between the two quantities. Of the interactions among 30 ERDEG, a strong correlation was observed between *SLC26A3* and *CLCA4* (**Figure 4D**). A heatmap was constructed to show a difference in the gene expression pattern in the patients with COAD and normal individuals (**Figure 4B**). A significant difference in the expression of 25 ERDEGs was observed in normal individuals and patients with COAD, as shown by the boxplots (**Figure 4C**).

### Construction of enterocytes associated prognostic risk model based on TCGA training cohort

**Figure 5:**
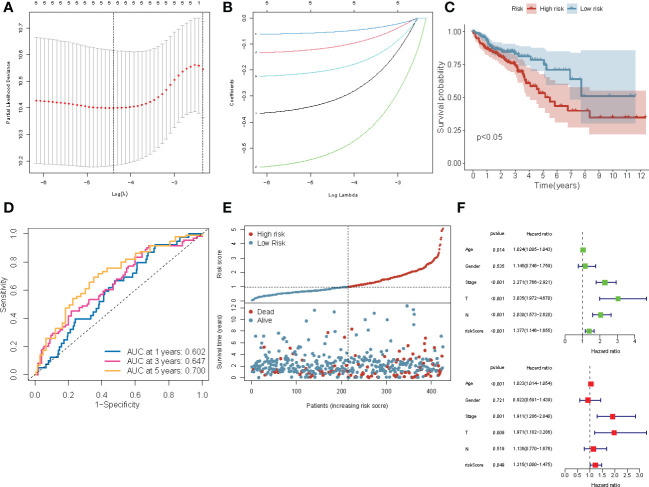
Construction of the four-gene prognostic risk model based on the TCGA-COAD cohort. **(A)** Screening of optimal parameters (lambda) in the LASSO regression model based on the TCGA cohort. **(B)** LASSO coefficient profiles of the 4 ERDEGs determined by the optimal lambda. **(C)** The Kaplan-Meier survival curve for the OS of patients with COAD patients in the high- and low-risk groups from TCGA cohort. **(D)** The ROC curve of the prognostic model shows the OS and survival status in TCGA cohort. **(E)** Scatterplots at the top and bottom show the distribution of the risk score and survival status in patients with COAD, respectively. **(F)** Univariate and multivariate Cox regression analyses of the risk score and clinicopathological parameters in TCGA cohort.

To identify differences in the gene expression patterns in patients with COAD, we subsequently investigated the possibility that creating a risk profile for enterocytes could aid in prognosis prediction. Univariate Cox regression analysis revealed a significant correlation (P< 0.05) between five ERDEGs (*CPM, CLCA4, ELOVL6, ATP2A3*, and *VSIG2)* and the overall survival (OS) of patients in the training cohort (**Supplementary Figure 1**). Based on these results, LASSO regression analysis was performed on the expression values of five ERDEGs to determine their ability to predict the prognosis (**Figures 5A, B**). *CPM, CLCA4, ELOVL6*, and *ATP2A3* were finally chosen to construct the four-gene signature risk prediction model.

The following formula was used to construct the four-gene signature prognostic risk model was


$$\begin{array}{c} Riskscore=(–0.3989\times expressionof\;CPM)\\ \;\;\;\;+(–0.1712\times expressionof\;CLCA4)\\ \;\;\;\;+(–0.5605\times expressionof\;\;ELOVL6)\\ \;\;\;\;+(–0.2608\times expressionof\;ATP2A3) \end{array}$$

(**Supplementary Table 4**) The risk scores of all patients in the TCGA training cohort were calculated, and then the patients were divided into high- and low-risk groups based on the median risk score. The Kaplan-Meier survival curve showed that the prognosis of patients with COAD in the high-risk group was worse compared to the low-risk group (P< 0.0001; **Figure 5C**). The area under the ROC curve (AUC) value for 5-year was 0.700; for three year was 0.647, and for one year was 0.602, thereby indicating that the prognostic model showed a satisfactory degree of accuracy in predicting survival (**Figure 5D**). **Figure 5E** shows the risk scores distribution and patient survival status. Univariable and multivariable Cox regression analyses were performed to determine if the model based on the risk score could be an independent predictor of OS. Univariate regression analysis showed a significant correlation (P< 0.05) between age, the AJCC stage, risk score, and OS. Similarly, multivariate Cox regression analysis showed that the age (P< 0.001), stage (P = 0.001), T (P = 0.009), and risk score (P = 0.049) could predict the prognostic of patients (**Figure 5F**). These results demonstrated that the risk score is an independent prognostic factor for patients with COAD.

### Internal and external validation of the four-gene signature prognostic risk model

**Figure 6:**
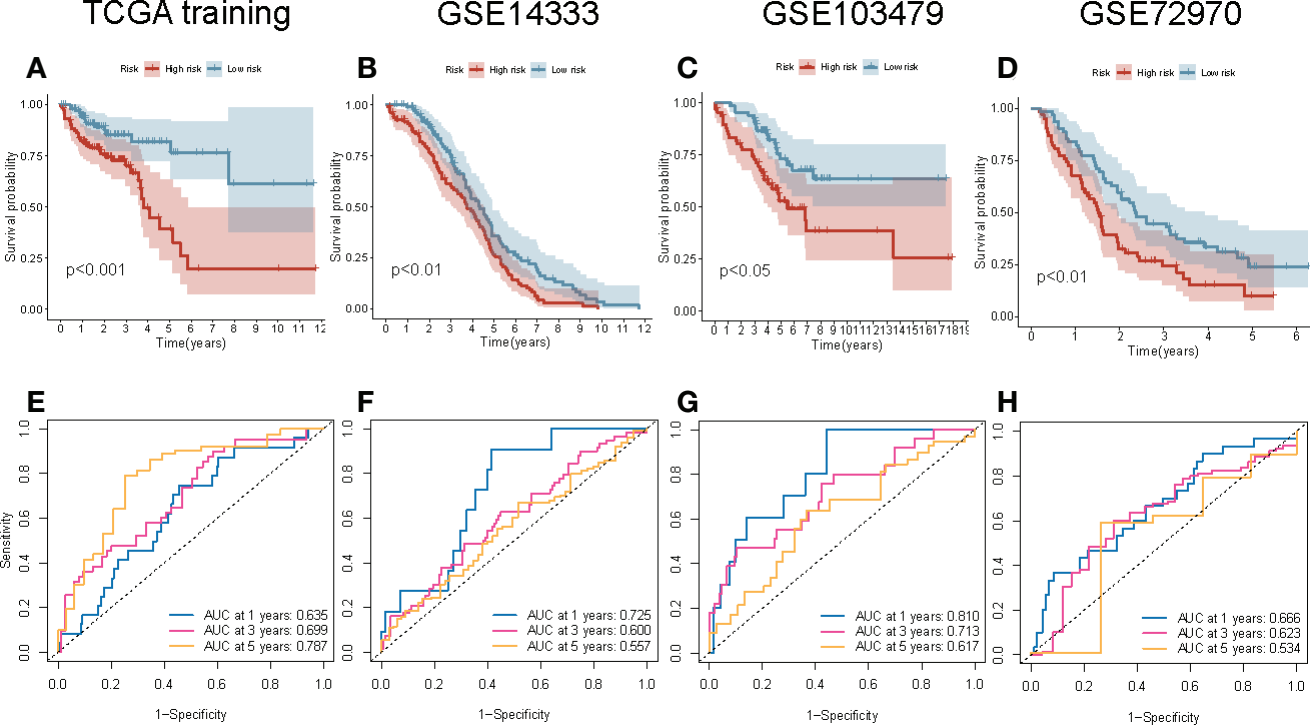
Internal and external validation of the four-gene signature prognostic risk model. **(A–D)** The Kaplan–Meier survival curve of the OS of patients with high-risk and low-risk scores in TCGA training, GSE14333, GSE103479, and GSE72970 cohorts. **(E–H)** The ROC curve of the prognostic model in TCGA training, GSE14333, GSE103479, and GSE72970 cohorts.

To determine the reproducibility and robustness of the four-gene signature prognostic risk model across different populations, the model was validated on the internal TCGA training cohorts and external validation cohorts. In the internal TCGA training cohort, the Kaplan-Meier survival curve showed that the OS of the patients with high-risk scores was shorter (P = 0.001; **Figure 6A**). Similarly, the AUC value of 1-, 3-, and 5-year survival rates for the internal TCGA training cohort were 0.635, 0.699, and 0.787, respectively. This indicates that the model had excellent performance in predicting the survival of patients (**Figure 6E**). Next, the external validation cohort included samples from the GSE14333, GSE10347, and GSE72970 datasets to validate the robustness and validity of the model. In the external validation cohort, the Kaplan-Meier survival curve revealed that the OS of the patients in the high-risk group was significantly shorter (**Figures 6B–D**). These results were consistent with the results of the internal TCGA training cohort. The results of the ROC curve of the three external validation cohorts were equivalent to the results of the above model (**Figures 6F–H**). Overall, the risk scores could discriminate across all four cohorts.

### Prognostic risk scores related to immune cell infiltration and immunotherapy response

**Figure 7:**
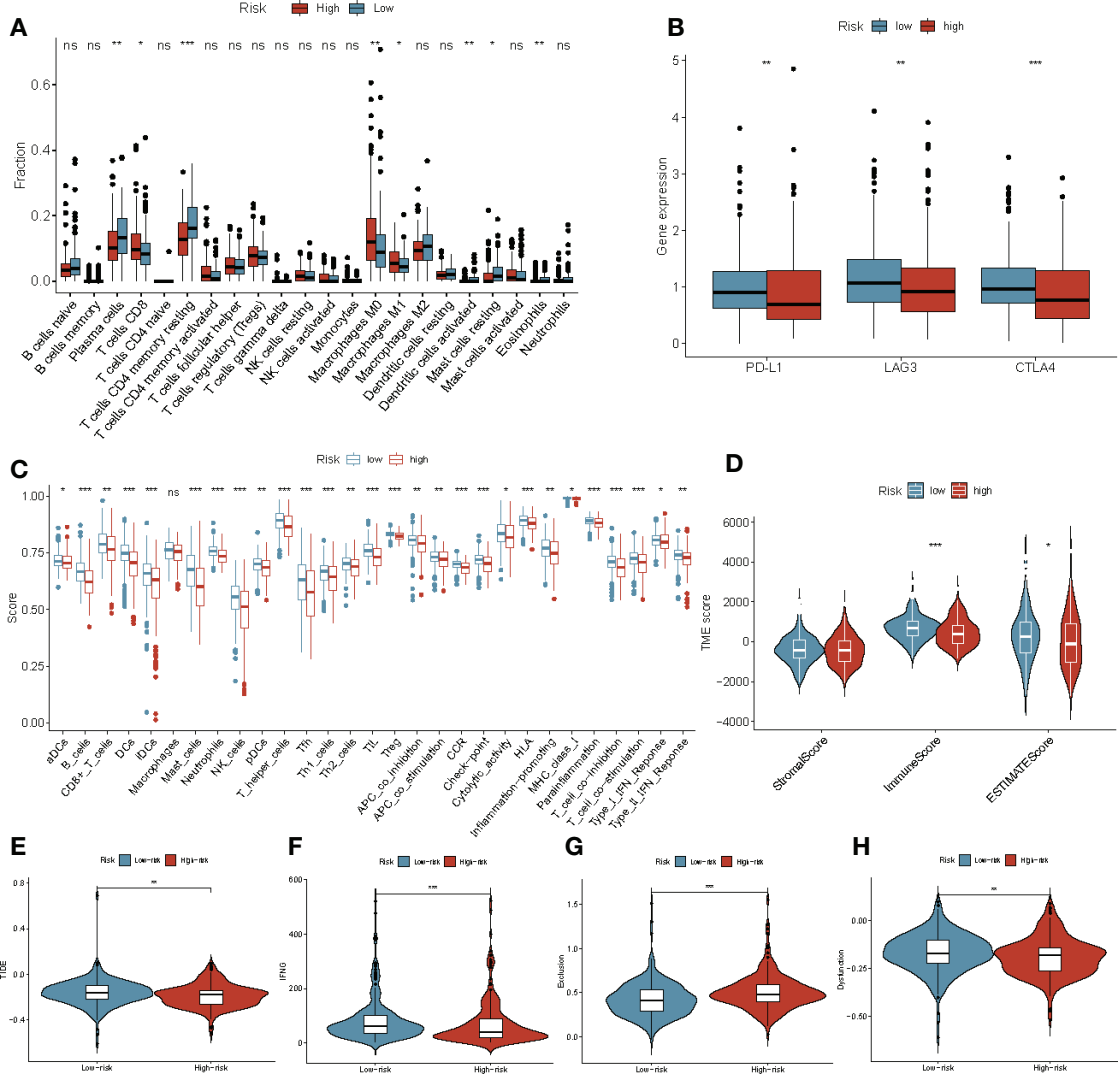
Immune landscape and immune status in patients with COAD in the high‐ and low‐risk groups. **(A)** The boxplots for the comparison of the 22 immune cells in patients with COAD in the high-risk and low-risk groups. **(B)** ICI-related genes between the high‐ and low‐risk groups. **(C)** ssGSEA was used to estimate the immune cell infiltration score and immune-related pathways enriched in the high- and low-risk groups. **(D)** The stromal score, ESTIMATE score, and immune score in patients with COAD in the high- and low-risk groups. **(E–H)** The Violin plot shows the TIDE **(E)**, IFNG **(F)**, T cell exclusion **(G)** and T cell dysfunction **(H)** scores in the high- and low-risk groups in TCGA-COAD cohort. *P< 0.05, **P< 0.01, ***P< 0.001, ns, not significant.

To understand the association between ERDEGs, immune cell infiltration, and lymphocyte receptor diversity in patients with COAD, the correlation between the four-gene signature and immune cell infiltration was first investigated. High levels of T-cells, M0, and M1 macrophages were observed in patients with COAD in the high-risk group. The levels of the plasma cells, resting memory CD4 T cell, activated dendritic cells, resting mast cells, and eosinophils were low in patients with COAD in the high-risk group (**Figure 7A**). Single-sample GSEA (ssGSEA) was used to evaluate the relative quantity of 29 immune markers to determine the status of immune cell infiltration in the two groups. By comparing box charts between groups, the immune infiltration landscape of the TCGA cohort was depicted in greater detail. The scores of all markers in the high-risk group were low compared to the low-risk group. (**Figure 7C**). The estimation of stromal and immune cells in malignant tumor tissues using Expression data (ESTIMATE) algorithm was used to calculate the ESTIMATE, immune, and stromal scores for both groups. Interestingly, the ESTIMATE and immune scores of the patients in the low-risk group were significantly higher (P< 0.05 and P< 0.001, respectively) compared to the patients in the high-risk group (**Figure 7D**). Therefore, we further assessed the correlation between the ERDEGs and immunotherapy response by analyzing the gene signatures and widely recognized immunotherapy biomarkers in TCGA cohort. Considering the important role of ICI-related genes in regulating immune response, we compared the expression of *PD-L1, LAG3*, and *CTLA4* in the patients in the high and low-risk groups. The results revealed that most ICI-related genes were expressed in the patients in the low-risk groups (**Figure 7B**). We determined the TIDE, T cell dysfunction, IFN-γ, and T cell exclusion scores to get a comprehensive overview. The IFN-γ scores (P< 0.001) were significantly low, and T cell exclusion scores (P< 0.001), TIDE score (P< 0.05), and T cell dysfunction scores (P< 0.01) were significantly high in the patients in the high-risk group (**Figures 7E–H**). This indicates that the patients in the high-risk group were more sensitive to immunotherapy. Taken together, these results suggest that patients with high-risk scores are more likely to benefit from immunotherapy, and the ERDEGs could serve as useful biomarkers to identify patients with COAD who may benefit from immunotherapy.

### Correlation between the immune status, cell types, and risk score

**Figure 8:**
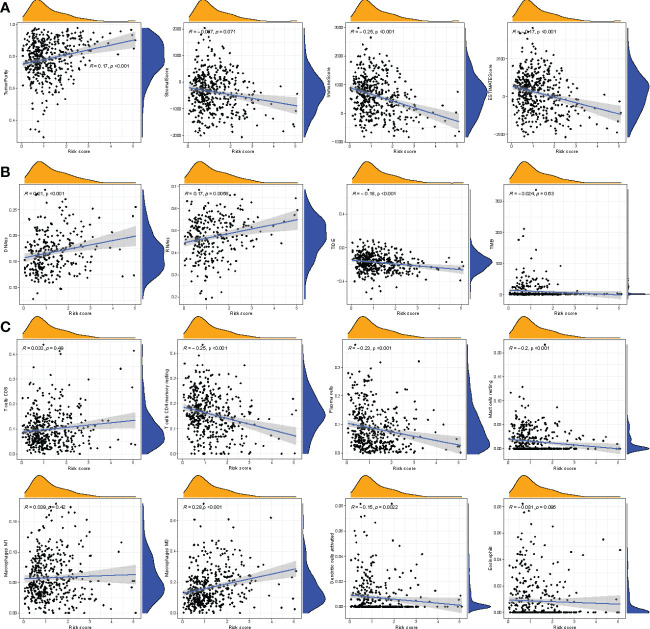
Correlation between riskscore and other scores. **(A)** Correlation between riskscore and tumor immune microenvironment. **(B)** Correlation between riskscore and stem cell scores, TIDE, tumor mutation burden. **(C)** Correlation between riskscore and immune cell infiltration-related cells.

We determined the correlation between the immune score, stromal score, ESTIMATE score, tumor purity, and risk score. A significantly negative correlation was observed between immune score, ESTIMATE score, and risk score, whereas a significant positive correlation was observed between risk score and tumor purity (**Figures 8A, B**). In addition, the correlation between infiltration of immune cell type and risk score was analyzed. As shown in **Figure 8C**, a positive correlation between the risk score and macrophages was observed, but a negative correlation was observed between plasma cells, dendritic cells, resting mast cells, resting memory T cells, and risk score. These results suggest that the four-gene signature prognostic risk model could accurately predict the status immune microenvironment of patients with COAD.

### Establishment and evaluation of the prognostic nomogram

**Figure 9:**
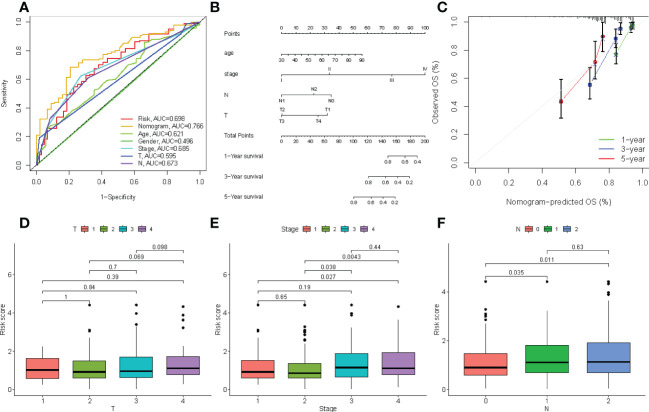
**(A)** Comparison of predictive performances of the four-gene signature prognostic risk model and other clinical characteristics. **(B)** The four-gene signature-based nomogram predicts 1-, 3-, and 5-year survival. **(C)** The calibration plot of the nomogram predicted and real surviving proportions. **(D–F)** The differences between riskscore and clinical characteristics.

The correlation between the clinical characteristics and risk scores was evaluated to validate the clinical utility of the four-gene signature prognostic risk model. As shown in **Figure 9A**, the AUC value for risk score was 0.698, the nomogram was 0.766, the age was 0.621, the stage was 0.685, and N was 0.698 in TCGA cohort. Our results revealed that the risk scores and profiling were more effective in predicting prognosis than individual clinical characteristics. In addition, a nomogram was created to predict 1-, 3-, and 5-year survival of patients with COAD. Univariate and multivariate COX regression analyses were performed on the risk scores for patient characteristics, age, and tumor stage (**Figure 9B**). The C-index of the nomogram was 0.6031, and the calibration plot was also drawn to demonstrate the predictive accuracy of the nomogram, with observations at 1, 3, and 5 years relative to predicted observations (**Figure 9C**). As shown in **Figures 9D–F**, we determined the predictive value of the risk score, which was believed to be related to the AJCC stage.

### The expression of signature genes in normal, polyp, and COAD tissues

**Figure 10:**
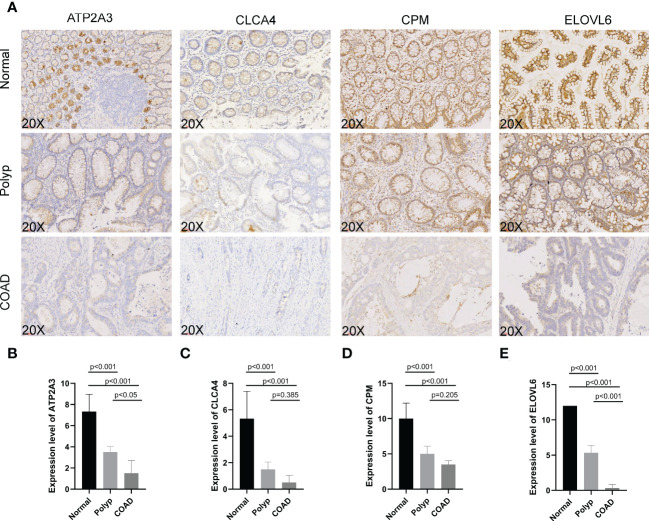
**(A–E)** Immunohistochemistry was performed to study ATP2A3, CLCA4, CPM, and ELOVL6 expression in tissues from normal individuals, patients with polyps, and COAD.

IHC was used to determine the expression of signature genes (*CPM, CLCA4, ELOVL6*, and *ATP2A3*) in tissues from patients with COAD for validating their accuracy. Tissues from six normal individuals, four patients with polyps, and six patients with COAD were used to determine the expression pattern of four genes. Four slides/sample was obtained to perform IHC (**Figure 10A**). The expression of CPM, CLCA4, ELOVL6, and ATP2A3 was high in tissues from normal individuals. Moderate expression of CPM, CLCA4, ELOVL6, and ATP2A3 was observed in tissues from patients with polyps. The expression of these genes was low in the tissues of patients with COAD. A significant difference (P< 0.001) in the expression of all four genes was observed in tissues from normal individuals and patients with polyps and COAD. A significant difference in the expression of *ELOVL6* and *ATP2A3* was observed in tissues of patients with polyp and patients with COAD (P< 0.001 and P< 0.05, respectively, **Figures 10B–E**). These results suggest that these signature genes could confer protection against COAD progression. In addition, *ELOVL6* and *ATP2A3* could be used as markers for progression from polyps to COAD.

### Drug sensitivity analysis between the low- and high-risk groups

The “pRRophetic” R package was used to compare the estimated IC_50_ value of small molecule drugs in the high-risk and low-risk groups for determining the difference in drug resistance in patients in the two groups. A significant difference in the sensitivity to elesclomol, shikonin, and bryostatin-1 was observed between the patients in the two groups (P< 0.001; **Supplementary Figure 2**).

## Discussion

The 5-year survival rate for patients with colon cancer diagnosed at an early stage is approximately 90%; however, the survival rate declines to 14–15% in patients with distant metastasis (stage IV) (17). Hence, the identification of novel and potent prognostic biomarkers is essential for preventing the progression of the disease. The precursor adenomatous polyps are the main cause of most colon cancers. The acquired and inherited genetic mutations cause malignant transformation of the epithelial cells. More than 80% of human cancers, including COAD, originate from epithelial cells (18). Becker WR et al. observed an increasing number of epithelial cells occupy a stem cell-like state at the transcript level through snRNA-seq and scATAC-seq (19). We further investigated the differences of cell type among samples from COAD patients by scRNA-seq. Compared to scRNA-seq, snRNA-seq reveals the nuclear RNA inflammation and scATAC-seq DNA fragment does not have a PolyA tail. In this study, we further investigated the differences in the cell types in the tissues of patients with COAD. The results showed that the epithelial cells exhibited a growing stem cell-like phenotype from normal to the polyp stage, and the enterocytes play a key role in mediating communication in the epithelial cells. This could be due to the abnormal expression pattern of some pro-oncogenes in enterocytes. Based on these results, we used publicly available databases to obtain datasets on the scRNA-seq for identifying DEGs in enterocytes during the transformation from normal to polyps to adenocarcinoma in patients with COAD. Compared to normal individuals, EMT was upregulated in patients with COAD. Various studies have shown that EMT plays a role in the migration and invasion of COAD cells and is an important pathological process contributing to cancer progression. The EMT markers could serve as prognostic biomarkers and potential therapeutic targets for the treatment of colon cancer (18, 20). Based on these ERDEGs, in this study, we constructed a unique four-gene signature prognostic model that could be an independent risk factor. This four-gene prognostic risk model was validated in internal and external validation cohorts.

Several studies have investigated the efficacy of targeting immunosuppressive cells to boost antitumor immunity in preventing the progression of precancerous adenomas to CRC (21). Currently, the expression of genes like *CD274, LAG3, CTLA4*, and TIDE score is widely used to predict the patient’s response to ICI treatment. ICI drugs can enhance immune function by promoting T cell death *via PD-L1* and IFN-γ. TIDE score has been used to predict response to immunotherapy recently, and the accuracy of the TIDE score in predicting immunotherapy outcomes is better compared to other biomarkers or indicators. Therefore, to study the difference in the patient’s response to immunotherapy between the two groups, the expression of *CD274, LAG3, CTLA4*, and the TIDE scores were determined in patients in the high-risk and low-risk groups. Our results show a positive correlation between low-risk scores and many immunotherapy biomarkers. Therefore, identifying patients with high-risk scores at the pre-malignant stage could aid in improving their response to ICI treatment. The biological pathways and immunological profiles were analyzed, and the results revealed that the gene signature enriched various well-known oncogenic pathways. The patients with a low-risk score were identified by their immune cell levels and immune activity.

In our study, we constructed a four-gene signature prognostic model based on enterocyte-related marker genes like *CPM, CLCA4, ELOVL6*, and *ATP2A3*. *ATP2A3* encodes the SERCA pump, which pumps Ca2+ into the lumen of the endoplasmic reticulum. A study has shown that *SERCA3* expression was regulated by the proximal promoter of *ATP2A3* during the induction of epithelial cancer cell differentiation (22). Previous studies have shown that the expression of *SERCA3* is low or not expressed by colon cancer cell lines (23). Therefore, high SERCA3 expression in the stomach and colon cancer could serve as a prognostic biomarker. Brouland et al. showed a negative correlation between *SERCA3* levels and poor epithelial cell differentiation. Further, *SERCA3* expression was low in adenocarcinomas (24). These results suggest a close correlation between the transition from adenomatous polyps to adenocarcinoma, CRC development, and the aberrant *SERCA3* expression. Carboxypeptidase-M (*CPM*) is a cell membrane-bound peptidase expressed by various cells like trophoblasts and alveolar epithelia. *CPM* can metabolize bioactive peptides, hormones, and cytokines (25). In recent years, *CPM* has been identified as a potential biomarker for cancers (26). Recent studies have shown that *CPM* was a direct target of miR-146a-5p. The binding of MIR146A5p to *CPM* inhibits the migration and invasion of CRC cells by regulating the expression of *SRC* and *FAK* (27). *CLCA4* is a tumor suppressor and a member of the calcium-activated chloride channel protein family, which is associated with the growth, proliferation, migration, and invasion of tumor cells. A study has shown decreased expression of *CLCA4* in colon cancer, which was associated with the development and progression of colon cancer (28). Mo et al. showed that 11.8% of patients with CRC have high microsatellite instability and harbor *CLCA4* frameshift mutations in repeat sequences (29). MIR19a regulates the growth, migration, and invasion of CRC cells by decreasing *CLCA4* expression (30). Studies have shown that *CLCA4* inhibits cell proliferation and metastasis in bladder cancer (31) and liver cancer (32) *via* the PI3K/AKT signaling pathway.

We have performed IHC to determine the expression of these four genes in tissues from normal individuals, patients with polyps, and patients with COAD. These results were consistent with previous studies; therefore, it is tempting to postulate that *CPM, CLCA4*, and *ATP2A3* could confer protection against COAD. *ELOVL6* is an endoplasmic reticulum enzyme that catalyzes the synthesis of long-chain fatty acids. Studies have shown an association between fatty acid metabolism and the development of colon cancer (33). Tissues from patients with colon cancer have enhanced fatty acid elongation, and lipid metabolism plays a role in the transition from colorectal polyps to CRC (34, 35). *ELOVL6* could be a therapeutic target for multiple cancers, and high *ELOVL6* expression was associated with poor prognosis in patients with breast cancer (36). Knockdown of *ELOVL6* expression inhibited hepatocellular carcinoma cell proliferation *in vitro* and *in vivo* (33). Meanwhile, a positive correlation was observed between high *ELOVL6* expression and the survival of patients with hepatocellular carcinoma (37). Our IHC results showed that the expression of ELOVL6 was low in the tissues of patients with COAD.

However, our current study has a few limitations. Additional studies are required to determine whether the four genes can distinguish precancerous adenoma from its benign counterpart. In this study, we retrieved data from several publicly available databases to construct the four-gene signature prognostic model. However, large-scale prospective clinical trials are required to confirm the accuracy of the prognostic model in predicting survival. Moreover, the IHC results showed no difference in the expression of CPM and CLCA4 in tissues from the patients with polyp and COAD, which could be due to the quantity of samples.

In conclusion, we used single-cell and bulk RNA-sequencing to construct and validate a novel four ERDEGs signature prognostic model. This model could serve as a potential prognostic biomarker and predict immunotherapy responses in patients with COAD. Our study sheds new light on the role of immune cell marker genes in prognosis and immunotherapy response in patients with COAD.

## Data availability statement

The original contributions presented in the study are included in the article/**Supplementary Material**. Further inquiries can be directed to the corresponding authors.

## Ethics statement

The studies involving human participants were reviewed and approved by the ethics committee of Huadong Hospital Affiliated to Fudan University (Shanghai, China; No.2019K069). All patients signed informed consent forms provided by the hospital. The patients/participants provided their written informed consent to participate in this study. Written informed consent was obtained from the individual(s) for the publication of any potentially identifiable images or data included in this article.

## Author contributions

XC and JL conceived and designed the study. XC and YF drafted and revised the manuscript. YW and LH assisted with the manuscript review. All authors contributed to the article and approved the submitted version.

## Funding

The work was funded by the General Hospital Science and the research fund of the Shanghai Municipal Planning Commission (No. 17dz2307500), Shanghai TCM special department (special disease) alliance project (HWZG (2021) No. 13), TCM Scientific Research Project of Shanghai National Health Commission (No. 2022QN017).

## Conflict of interest

Author YW was employed by GeneScience Pharmaceuticals Co. Ltd.

The remaining authors declare that the research was conducted in the absence of any commercial or financial relationships that could be construed as a potential conflict of interest.

## Publisher’s note

All claims expressed in this article are solely those of the authors and do not necessarily represent those of their affiliated organizations, or those of the publisher, the editors and the reviewers. Any product that may be evaluated in this article, or claim that may be made by its manufacturer, is not guaranteed or endorsed by the publisher.
