# Supplementary material for: A novel enterocyte-related 4-gene signature for predicting prognosis in colon adenocarcinoma
Source: Front Immunol. 2022 Dec 2;13:1052182. doi: 10.3389/fimmu.2022.1052182 (PMC9755665; doi:10.3389/fimmu.2022.1052182)
Supplement: Supplementary file 1 [file DataSheet_1.docx]

Supplementary Material

# Supplementary Figures and Tables

## Supplementary Tables

| **Variables** | **TCGA-COAD** | **GSE14333**  **(Validation1)** | **GSE103479**  **(Validation2)** | **GSE72970**  **(Validation3)** |
| --- | --- | --- | --- | --- |
| **Total** | 426 | 223 | 154 | 124 |
| **Gender** |  |  |  |  |
| Female | 198 | 105 | 68 | 50 |
| Male | 228 | 118 | 86 | 74 |
| **Age** |  |  |  |  |
| <60 | 120 | 60 | 28 | 52 |
| ≥60 | 306 | 163 | 124 | 72 |
| **AJCC stage** |  |  |  |  |
| I | 73 | - | 0 | - |
| II | 165 | - | 82 | - |
| III | 119 | - | 72 | - |
| VI | 58 | - | 0 | - |
| Unknow | 11 | - | 0 | - |
| **T stage** |  |  |  |  |
| T1 | 10 | - | 1 | 1 |
| T2 | 74 | - | 6 | 7 |
| T3 | 291 | - | 109 | 50 |
| T4 | 50 | - | 38 | 37 |
| Tis | 1 | - | 0 | 29 |
| **M stage** |  |  |  |  |
| M0 | 317 | - | 85 | - |
| M1 | 58 | - | 0 | - |
| Mx | 44 | - | 69 | - |
| Unknow | 7 | - | 0 | - |
| **N stage** |  |  |  |  |
| N0 | 253 | - | 82 | 14 |
| N1 | 99 | - | 50 | 28 |
| N2 | 74 | - | 22 | 53 |

# Supplementary Table 1.Demographic information of COAD patients in the present study.

| Cell Marker | Cell Type |
| --- | --- |
| PTPRC | T cell |
| CD163 | Macrophages |
| FGF7 | Fibroblasts |
| VWF | Endothelial cell |
| EPCAM | Epithelial cell |
| S100B | Enteric glial cell |
| KIT | Mast cell |
| CD44 | Cancer cell |
| MKI67 | Transit amplifying cell |
| LGR5 | LGR5+ stem cell |
| KRT20 | Enterocytes |
| MUC4 | Goblet cell |

# Supplementary Table 2 Classical Cell Markers.

| Number | Gene | Number | Gene |
| --- | --- | --- | --- |
| 1 | CKB | 16 | MUC4 |
| 2 | PDE3A | 17 | EFNA5 |
| 3 | FKBP5 | 18 | DUOXA2 |
| 4 | SCNN1B | 19 | KIAA0319 |
| 5 | XAF1 | 20 | AC073050.1 |
| 6 | SYTL2 | 21 | ELOVL6 |
| 7 | DUOX2 | 22 | EGLN3 |
| 8 | CPM | 23 | PID1 |
| 9 | RNF43 | 24 | NKD1 |
| 10 | NR6A1 | 25 | VSIG2 |
| 11 | B3GALT5 | 26 | CDHR5 |
| 12 | CLCA4 | 27 | ATP2A3 |
| 13 | PLPP1 | 28 | CFAP44 |
| 14 | AL589669.1 | 29 | AGBL4 |
| 15 | SLC26A3 | 30 | DDX60 |

# Supplementary Table 3. 30 enterocyte-related genes.

| Gene | Coef |
| --- | --- |
| CPM | -0.39892 |
| CLCA4 | -0.17124 |
| ELOVL6 | -0.56053 |
| ATP2A3 | -0.2608 |

# Supplementary Table 4. Multivariate COX regression analysis results of model genes.

## Supplementary Figures


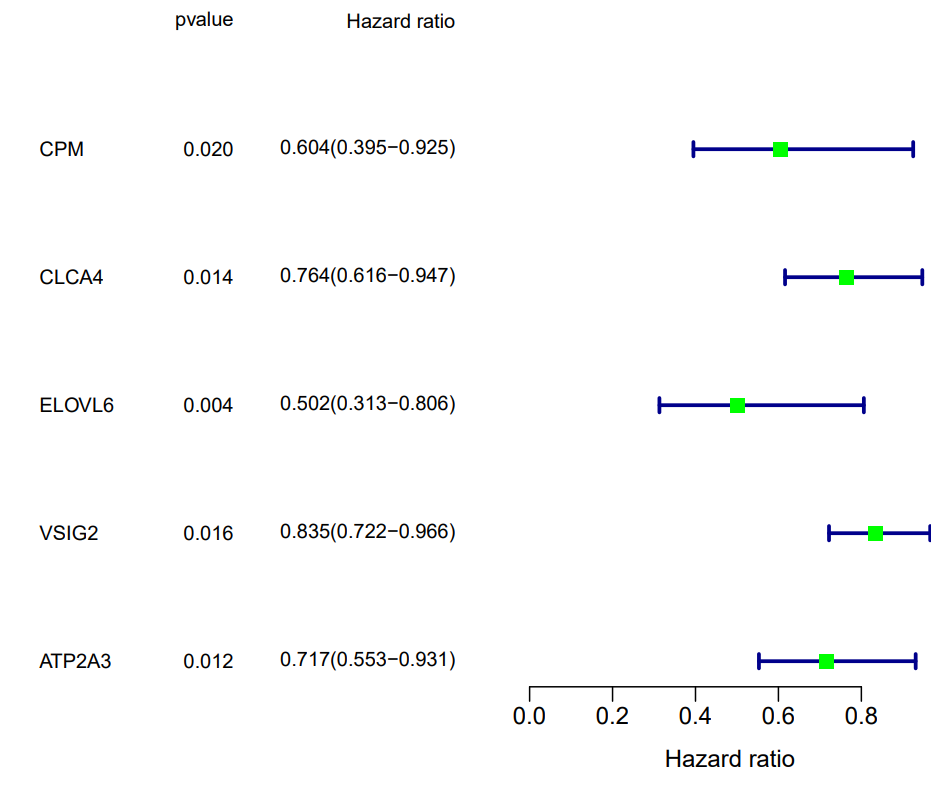


**Supplementary Figure 1.** Forest map of 5 ERGs significantly correlated with OS, identified by univariate cox analysis.


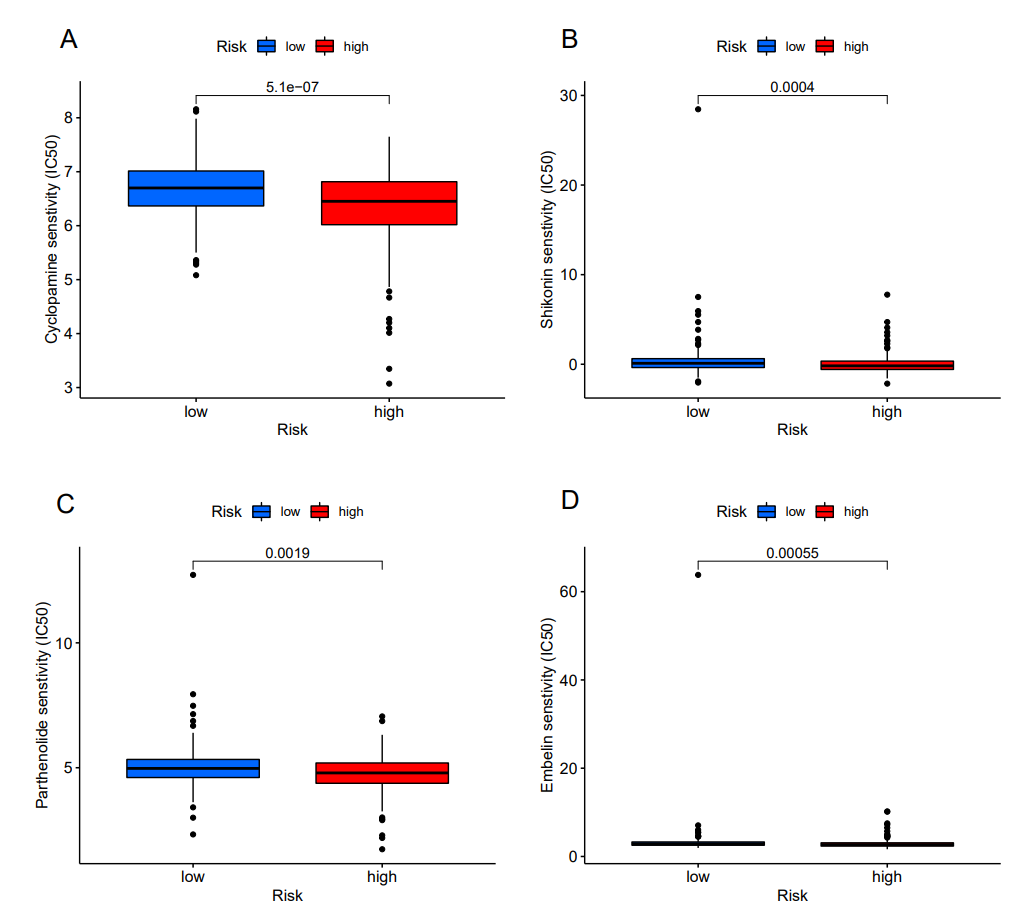


**Supplementary Figure 2.** Drug sensitivity analysis in the high-risk and low risk groups in COAD patients. (A) Cyclopamine (B) Shikonin (C) Parthenolideand and (D) Embelin.
